# Th17 Response in Uveitis: A Double-Edged Sword in Ocular Inflammation and Immune Regulation

**DOI:** 10.1007/s12016-025-09038-1

**Published:** 2025-03-12

**Authors:** Yuan Zong, Xue Tong, Wai Po Chong

**Affiliations:** 1 School of Chinese Medicine, Hong Kong Baptist University, Hong Kong, China; 2 Institute for Research and Continuing Education, Hong Kong Baptist University, Shenzhen, China

**Keywords:** Uveitis, Th17 response, IL-17, Autoimmunity

## Abstract

Uveitis involves a complex interplay of immune cell infiltration and cytokine imbalances, with Th17 cells playing a central role in this process. Th17 cells contribute to disease pathogenesis by promoting inflammation, recruiting additional immune cells, and directly damaging retinal tissues. This review discusses the current knowledge on therapeutic strategies targeting Th17-related cytokines, including cytokine blockade, small molecule inhibitors, and immunomodulatory approaches. Traditionally, Th17-related cytokines have been viewed as pro-inflammatory agents in uveitis. However, emerging research has highlighted the capacity of the Th17 response to express immunoregulatory cytokines, notably IL-10, IL-24, and TGF-β. This suggest that the Th17 response may have a dualistic role that includes immune suppression. In this review, we will discuss this paradoxical nature of Th17 cells in immune regulation and inflammation that they can both promote and mitigate uveitis. We expected that a deeper understanding of these mechanisms is imperative for the innovation of novel therapeutics that could consider the dual role of Th17 response in the pathogenesis of uveitis. By finely tuning the Th17 response to preserve retinal integrity and function, these new treatments could bring significant benefits to patients with uveitis. This review aims to shed light on the complexities of the Th17 response in uveitis and its implications for future therapeutic strategies.

## Introduction

Uveitis encompasses a spectrum of intraocular inflammatory conditions targeting that mainly the uvea, i.e., iris, ciliary body and choroid, as well as adjacent structures, including retina and vitreous [1]. It arises from an inappropriate activation of the immune system, leading to ocular inflammation that, if left unchecked, can result in irreversible damage to the sensitive components of the visual system [2]. These conditions represent significant global health burdens, contributing to visual impairment and blindness, particularly in developing nations. Understanding the mechanisms underlying ocular inflammation and its association with these ocular diseases is crucial for the development of effective therapeutic interventions.

Th17 cells, the IL-17 producing CD4^+^ T helper cells, are formed by heterogeneous subsets with different regulations and functions and have gained significant attention due to their crucial role in immune-mediated diseases, including uveitis [2–5]. Th17 cells are characterized by the production of signature cytokines including interleukin (IL)−17, IL-17F, IL-21, and IL-22 and the expression of the master transcription factor, retinoic acid–related orphan receptor (ROR)- γt [6]. Under specific microenvironment, they secrete addition proinflammatory cytokines, such as granulocyte–macrophage colony-stimulating factor (GM-CSF) [7, 8] and interferon (IFN)-γ [9] for tissue damage. Originally identified as key players in host defense against extracellular pathogens, Th17 cells and their signature cytokines have since been implicated in the pathogenesis of various autoimmune and inflammatory disorders, including ocular autoimmune diseases [2, 10].

In the context of autoimmune uveitis, Th17 cells have emerged as important mediators of immune dysregulation [11, 12]. These cells are involved in the initiation and perpetuation of inflammatory responses within the eye as demonstrated in patients with uveitis as well as the animal model, experiment autoimmune uveitis (EAU), which is usually induced by two retinal antigen, namely interstitial retinol–binding protein (IRBP) and S-antigen [2, 13]. Th17 cells are recruited to the site of inflammation in the eye, where they secrete pro-inflammatory cytokines, including IL-17A, IL-17F, GM-CSF, and IL-22 [11, 14, 15]. These cytokines contribute to the recruitment and activation of other immune cells, such as neutrophils and macrophages, and promote the production of additional inflammatory mediators [2, 14]. The activity of Th17 cells and their cytokines can lead to tissue damage and disruption of the blood-retinal barrier, further exacerbating ocular inflammation. Targeting Th17 cell–associated pathways and cytokines may offer new therapeutic possibilities for modulating ocular inflammation and improving visual outcomes in these conditions. Further research into the intricate interplay between Th17 cells and ocular inflammation will undoubtedly shed light on potential therapeutic strategies for managing ocular diseases with an inflammatory component [2].

In this review, we aim to provide a comprehensive overview of Th17 response in the pathogenesis of uveitis. We will discuss the underlying mechanisms of its cytokine milieu in ocular inflammation. Furthermore, we will explore potential therapeutic targets and interventions aimed at modulating Th17 response to prevent or treat autoimmune uveitis. Contrary to its conventional classification as a promoter of inflammation, some studies have unveiled a more nuanced role for Th17 cells. They appear to have the ability to produce immunoregulatory cytokines, particularly IL-10 and IL-24 [5, 9], and IL-17 increases the number of regulatory T cells (Tregs) [16]. This suggests that Th17 response may have a bifunctional role that encompasses both immune activation and suppression. By elucidating the intricate relationship between Th17 response and autoimmune uveitis, we hope to contribute to the development of innovative approaches for managing ocular autoimmune diseases and improving visual outcomes.

### Th17 Response and Its Role in Inflammation

Th17 response refers to the activation and expansion of CD4^+^ T helper cells that produce IL-17 and other pro-inflammatory cytokines. Th17 cells play a critical role in immune responses against pathogens and is involved in the pathogenesis of various inflammatory diseases. The primary function of the Th17 response is to provide protection against extracellular pathogens, particularly fungi and bacteria [17]. IL-17 and other cytokines produced by Th17 cells recruit and activate neutrophils, which are crucial for combating these types of infections [18]. Th17 cells also stimulate the production of antimicrobial peptides and chemokines, further enhancing the immune response at the site of infection. However, dysregulation of the Th17 response can contribute to the development of chronic inflammatory diseases. Excessive or sustained Th17 activation can lead to tissue damage and perpetuate inflammation. Aberrant Th17 responses have been implicated in autoimmune disorders such as rheumatoid arthritis, psoriasis, and inflammatory bowel disease [17]. In these conditions, Th17 cells infiltrate affected tissues and release pro-inflammatory cytokines, leading to immune cell recruitment, tissue destruction, and chronic inflammation. In addition to autoimmune diseases, the Th17 response has been implicated in other inflammatory conditions, including neuroinflammatory disorders, such as multiple sclerosis, and allergic diseases, such as asthma. Th17 cells and their cytokines can contribute to the breakdown of the blood–brain barrier in neuroinflammation and promote the recruitment of immune cells into the central nervous system [19]. In asthma, Th17 cells are involved in airway inflammation and the recruitment of eosinophils, contributing to bronchial hyperresponsiveness [20, 21]. Understanding the role of the Th17 response in inflammatory diseases is essential for developing targeted therapeutic strategies. Modulating Th17 cell activity or targeting Th17-associated cytokines may offer potential treatment options for managing these conditions [10]. However, it is important to note that the Th17 response is a complex and dynamic process, and further research is needed to fully unravel its intricacies and exploit the therapeutic potential of targeting Th17 response in inflammation.

### Th17 Cells: The Driving Force Behind Uveitis Pathogenesis

Human autoimmune uveitis has been associated with the presence of Th17 response as supported by the elevation of IL-17 serum level and the expression of IL-17 from peripheral blood mononuclear cell (PBMC) in patients with uveitis and more importantly, high IL-17 level is associated with active uveitis and disease severity [4, 22, 23]. In addition, a genome-wide study on Bechet’s disease has identified that regions encompassing IL-10 and IL23R-IL12RB2, which are highly involved in Th17 differentiation, are associated with disease susceptibility [24]. To further elucidate the role of Th17 cells in the pathogenesis of uveitis, researchers have turned to animal models. Experimental autoimmune uveitis (EAU) is commonly induced in these models by immunizing animals with a retinal protein and bacterial adjuvants, such as pertussis toxin and complete Freund’s adjuvant [2].

The pivotal discovery that IL-23, rather than IL-12, is essential for the development of autoimmune diseases in animal models [25–27], has shifted the focus towards the Th17 response. This shift underscores the importance of Th17 cells, rather than Th1 cells, as the principal drivers of EAU pathogenesis [4, 11]. Luger et. al. reported that targeting IL-17, the defining cytokine produced by Th17 cells, markedly reduced ocular inflammation in mice with EAU. Furthermore, their research demonstrated that merely transferring Th17 cells predisposed to induce uveitis is sufficient to penetrate the retinal barrier and trigger the onset of EAU [11]. A follow-up study underscored the pivotal role of Th17 cells in the development of EAU. The study revealed that conditional knocking out STAT3, a key transcription factor necessary for the differentiation of Th17 cells [28], in CD4^+^ cells led to a deficiency of IL-17-producing CD4^+^ T cells in mice immunized for EAU. Consequently, these mice did not develop the disease, highlighting the essential contribution of Th17 cells to the pathogenesis of EAU [29]. The activation of uveitogenic Th17 cells has been associated with overactivation of innate components, including macrophages, dendritic cells, neutrophils, γδT cells, and natural killer cells [30–32]. It has been well demonstrated that proinflammatory macrophages and dendritic cells expressed high level of IL-6, IL-23, and other co-stimulatory molecules to promote the pathogenic Th17 cells, while Th17 cells activate them reciprocally by the expression of GM-CSF [33]. In addition, the essential role of the gut microbiome has been highlighted in the induction and activation of retinal-IRBP-specific Th17 cells in the gut of R161H mice, which spontaneously develop EAU [34]. A subsequent study in patients with Behcet’s uveitis also confirmed that their gut microbiome has a higher potential to induce IL-17 production from their PBMCs [35]. These suggest the complexity of regulating Th17 response during the pathogenesis of uveitis.

Following the elucidation of Th17 cells’ involvement in EAU, research efforts have pivoted towards targeting the Th17 pathway to mitigate this condition. The differentiation of Th17 cells is contingent upon the presence of two pro-inflammatory cytokines: IL-6 and IL-23 [6]. Strategies that involve neutralizing these cytokines with antibodies or inhibiting their receptors have been demonstrated to alleviate EAU [11, 36, 37]. Later, IL-1β has also been identified as a critical factor in promoting the differentiation of pathogenic Th17 cells [38]. This pivotal role of IL-1β has similarly been observed in the context of EAU [39]. More cytokines have been reported to ameliorate EAU by limiting the differentiation of Th17 cells, e.g., IFN-γ, IFN-α2a, IFN-β, and IL-27 or inhibiting the effector functions of Th17 cells, e.g., IL-10, IL-21, IL-24, and IL-35 [5, 39–45]. These findings suggest that cytokine-based immunotherapy aimed at modulating the differentiation and activity of Th17 cells represent a potential avenue for the development of effective treatments for uveitis.

**Fig. 1:**
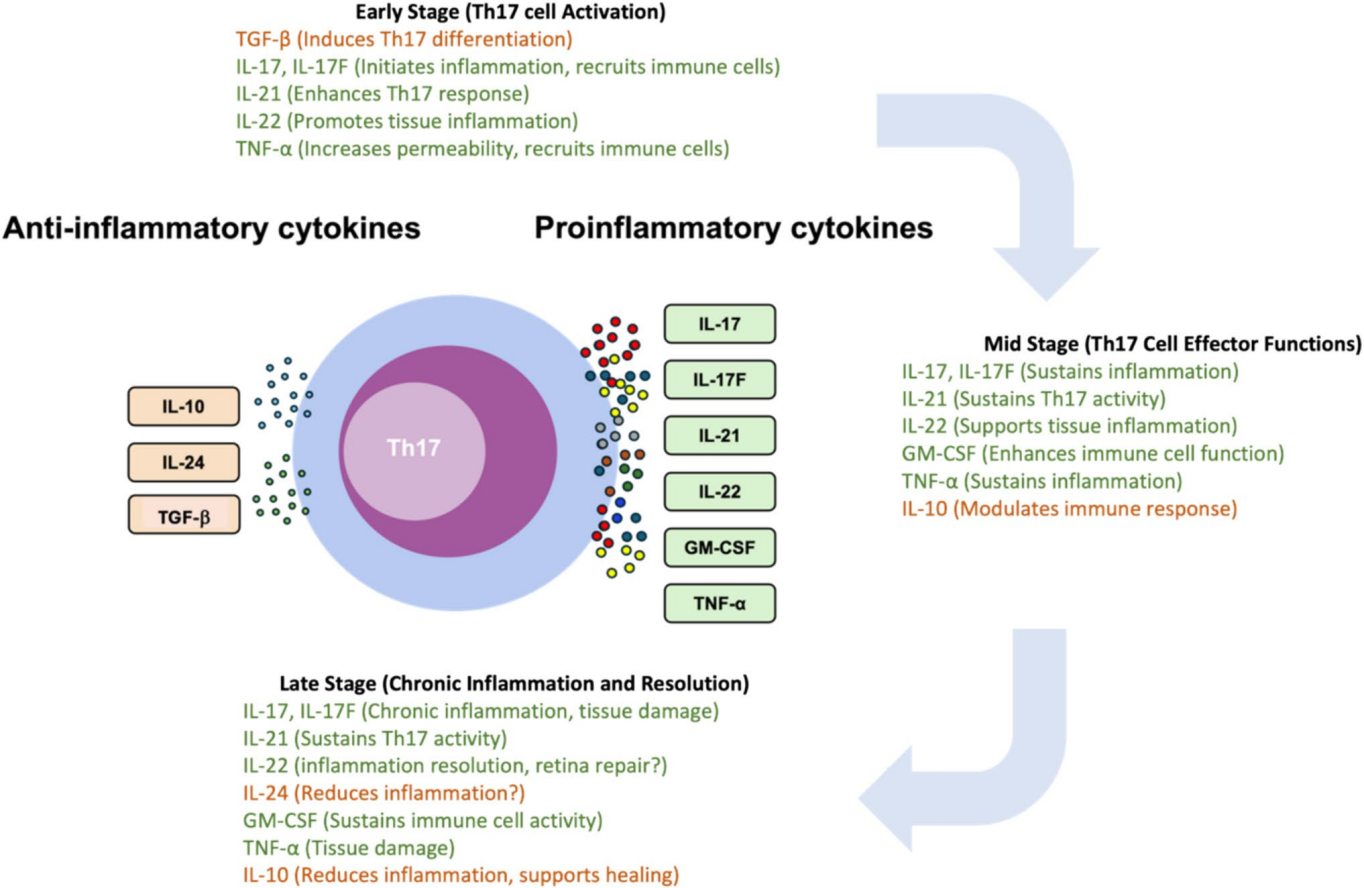
Cytokine profile of Th17 cells. Th17 cells are known to secrete pro-inflammatory cytokines that contribute to the pathogenesis of autoimmune conditions such as uveitis. Yet, these cells also have the capacity to produce anti-inflammatory cytokines, such as IL-10 and IL-24 and TGF-β, which have been associated with the resolution of immune responses

Th17 cells orchestrate inflammatory responses and tissue damage primarily through the secretion of proinflammatory cytokines, including IL-17, IL-17F, IL-21, IL-22, and GM-CSF [6] (Fig. 1). IL-17 and IL-17F are critical for mediating inflammation. Both IL-17 and IL-17F bind to the same IL-17RA/RC heterodimer receptor, although IL-17F has a much lower binding affinity and exhibits relatively weaker biological potencies compared to IL-17 [46]. Consequently, IL-17 is more prominently studied for its role in autoimmune diseases, including uveitis. They stimulate various cell types, including fibroblasts, endothelial cells, epithelial cells, and immune cells, to produce additional proinflammatory cytokines and chemokines for the recruitment of neutrophils and other immune cells to sites of inflammation [17]. In retina, IL-17 has been shown to promote proinflammatory cytokines production from retinal pigment epithelial (RPE) cells, astrocytes, and retinal microglia [17, 42, 47–49] and negatively affect retinal ganglion cells and other neuronal cells, potentially leading to vision impairment. In addition, IL-17 has implicated in the damage of blood-retinal barrier (BRB) by compromising the production of tight junction proteins from RPE cells and endothelial cells [19, 50].

IL-21, as one of the Th17 cytokines, is implicated in the pathogenesis of a spectrum of proinflammatory and autoimmune disorders [51]. Elevated levels of IL-21 have been detected in the serum and aqueous humor of patients diagnosed with uveitis, suggesting a role in ocular autoimmune processes [52]. In EAU, CD4^+^ T cells that produce IL-21 have been observed infiltrating the eyes during the development of the disease. Remarkably, attenuation of IL-21 expression in these cells has been demonstrated to reduce their pathogenic potential, leading to a decrease in ocular inflammation. This finding underscores the importance of IL-21 in the exacerbation of inflammatory responses within the eye [53].

IL-22, belonging to the IL-10 cytokine family, is recognized for its pivotal role in tissue regeneration and the defense of barrier surfaces [54]. Its regulatory functions are essential for maintaining the integrity of tissue barriers. Consequently, IL-22 has been associated with various conditions characterized by inflammatory tissue pathology [55]. Its role in uveitis, however, presents a paradox. Although there is a study reported the elevation of serum IL-22 level in patients with acute anterior uveitis [56] along with findings of IL-22-producing CD4^+^ Th cells in ocular specimens from patients with Behçet’s disease [57], several studies suggest a lack of positive correlation between IL-22 levels and uveitis, reporting either no association or a negative one [58–60]. Despite these conflicting observations in clinical settings, EAU studies have shed light on a potentially protective role for IL-22. IL-22 treatment has been shown to resolve ocular inflammation by promoting the regulatory CD11b^+^ antigen presenting cells (APCs) [60]. This protective mechanism aligns with findings from another research, indicating that IL-22 may act locally within the retina to diminish inflammatory damage by enhancing the suppressive functions of retinal glial Müller cells [61]. The precise function of IL-22 in the context of autoimmune uveitis remains to be fully delineated. Additional research is essential to characterize the cytokine’s exact mechanisms of action and its potential dual roles in this complex ocular condition.

GM-CSF plays an essential role in the development, maturation, and activation of immune cells, especially myeloid cells including neutrophils and monocytes/macrophages [62]. Consequently, it is implicated in the onset and progression of inflammatory and autoimmune disorders [62]. The importance of GM-CSF in Th17 cell–mediated neuroinflammation has been highlighted in experimental autoimmune encephalomyelitis (EAE), the animal model of multiple sclerosis. It has been shown that Th17 cells can instigate EAE through the action of GM-CSF alone, even in the absence of the traditionally recognized pro-inflammatory cytokines, IL-17 and IFN-γ [7, 8]. Elevated level of GM-CSF has also been reported in the aqueous humor of patients with uveitis, suggesting its role in causing retina inflammation [52, 63]. In the case of EAU, So et. al. reported that IL-17 and IFN-γ double knockout mice are still capable to develop full prone retina inflammation with the ocular infiltrates contained increased GM-CSF-producing CD4^+^ T cells, in which GM-CSF propels the disease pathology by activating and drawing eosinophils into the ocular tissue [64].

### Targeting Th17-related Cytokines in Uveitis

Strategies to mitigate Th17 cell–mediated inflammation have been developed, focusing on either inhibiting their differentiation and activation by depleting Th17-driving cytokines or neutralizing Th17 effector cytokines, as summarized in Table 1. The differentiation of Th17 cells from naïve CD4^+^ T cells is critically dependent on IL-23, IL-6, and IL-1β. Interventions aimed at these cytokines or their receptors have successfully suppressed Th17 cell proliferation and their cytokine production, thereby aiding the control of ocular inflammation in EAU [11, 12, 36, 37]. Ustekinumab, which blocks the Th1 and Th17 responses by targeting IL-12 and IL-23, has shown promise in treating spondyloarthritis-associated uveitis [65]. Additionally, Sarilumab, designed to inhibit Th17 cell differentiation by obstructing the IL-6 receptor, has been report to be effective in a phase 2 clinical trial for non-infectious uveitis [65, 66]. Anakinra, the IL-1 receptor antagonist, and canakinumab, the anti-IL-1β neutralization antibody, have shown improvement of retinal vasculitis lesions and uveitis flares with reduced steroid dosage in Behçet’s disease–related uveitis [67]. However, the efficacy of IL-17 neutralizing agents, such as ixekizumab and secukinumab, in treating uveitis has shown variable outcomes [10, 65, 68, 69]. Although ixekizumab display therapeutic effect on ankylosing spondylitis, it does not show any improvement in the related uveitis [70]. Three clinical trials of secukinumab in non-infectious uveitis did not meet the primary efficacy endpoints [71]. Collectively, these clinical studies suggest that while targeting Th17 cells is promising, depleting its signature cytokine, IL-17, yields discouraging outcomes. This indicates that IL-17 may have multiple roles in the pathogenesis of uveitis [10, 65, 68–71].

**Table 1:** Targeting Th17-related cytokines in uveitis

Target	Agent	Diseases	Clinical efficacy	Reference
IL-17	Ixekizumab	Psoriasis-associated uveitis, spondyloarthritis-associated uveitis	Partially effective Not effective	[68] [65]
IL-17	Secukinumab	Noninfectious uveitis, spondyloarthritis-associated uveitis	Not effective Not effective	[69, 71], [65]
IL-12/IL-23	Ustekinumab	Spondyloarthritis-associated uveitis	Partially effective	[65]
IL-6R	Sarilumab	Noninfectious uveitis	Effective	[65, 66]
IL-1b	Canakinumab	Behcet’s disease-related uveitis	Effective	[67]
IL-1R	Anakinra	Behcet’s disease-related uveitis	Effective	[67]

### An Immunoregulatory Role of Th17 Cells in Attenuating Immune Responses

The involvement of the Th17 response in the pathogenesis of autoimmune diseases, primarily through the secretion of proinflammatory cytokines, is well-established. However, a growing body of evidence suggests a more nuanced role for Th17 cells beyond their proinflammatory functions. Notably, Th17 cells have been shown to express immunoregulatory cytokines, including IL-10, IL-24, and transforming growth factor (TGF)-β (Fig. 1) [5, 9, 72–74]. Among these regulatory cytokines, IL-10 can directly feedback to Th17 cells and suppress their production of IL-17 and limit their pathogenicity in EAU [41]. Both TGF-β and IL-24 cannot directly inhibit the expression of IL-17 [5, 75]; instead, they achieve it indirectly. TGF-β is essential for Treg differentiation which in-turn suppresses Th17 cells to express IL-17 and limit the development of EAU [76, 77]. IL-24 induces Th17 cells to produce IL-10, which subsequently suppresses the production of IL-17 and ameliorate EAU [42, 74]. These direct and indirect mechanism highlights the complex interplay between cytokines in modulating Th17 cell activity and maintaining immune homeostasis. Intriguingly, the signature cytokine of Th17 cells, IL-17, has been implicated in the suppression of Th1-mediated colitis [78] and even the regulation of Th17 responses themselves [5, 79, 80], Additionally, IL-17 is reported to contribute to the induction of Foxp3^+^ regulatory T cells (Tregs) in the context of autoimmune uveitis [16].

The capacity of Th17 cells to produce the inhibitory cytokine IL-10 has been substantiated by multiple research findings. Zielinski et al. revealed that human Th17 cells, when primed with *Staphylococcus aureus*, are prompted to co-express IL-10, serving an immunoregulatory function [9]. Mechanistic studies revealed that the induction of IL-10 production in Th17 cells is mediated by the immunoregulatory cytokines TGF-β and IL-27 [72, 81]. The signaling pathways involved include Smad3/Smad4, which are activated by TGF-β, and c-Maf/RORγt/Blimp-1, which are engaged in response to IL-27 [72, 81]. These findings highlight a complex regulatory mechanism within Th17 cells that balances pro-inflammatory and anti-inflammatory functions. Subsequent transcriptional profiling aimed at unraveling the intricate regulatory networks governing the differentiation of mouse Th17 cells identified the expression of IL-10 during the later stages of their differentiation [82]. These IL-10-producing Th17 cells have then been found to be at the recovery phase of intestine inflammation and represents an intermediate state of Th17s transitioning into IL-10-producing type 1 regulatory T (Tr1) cells [83]. Consistent with these findings, IL-10-producing Th17 cells have been identified across various stages of EAU, extending from the initial onset through to the resolution phase, and persisting into the remission phase [84]. This evidence collectively suggests that IL-10 expression by Th17 cells is crucial for immunological downscaling following an inflammatory response.[83]. Consistent with these findings, IL-10-producing Th17 cells have been identified across various stages of EAU, extending from the initial onset through to the resolution phase, and persisting into the remission phase [84]. This evidence collectively suggests that IL-10 expression by Th17 cells is crucial for immunological downscaling following an inflammatory response.

IL-24, belonging to the IL-10 cytokine family, has been recently identified as an inhibitory cytokine secreted by Th17 cells [5, 74]. Initially characterized in Th2 cells, IL-24 has been demonstrated to exert regulatory influence over effector T cell functions [85]. In vitro studies elucidate the role of IL-24, showing its capacity to suppress the production of both IFN-γ and IL-17 in human CD4^+^ Th cells as well as CD8^+^ cytotoxic T cells [86]. These findings highlight the multifaceted nature of cytokine interactions and the complex immunoregulatory networks that maintain immune homeostasis [86]. In a transcriptomic analysis, IL-24, alongside IL-10, was detected during the late stages of Th17 cell differentiation, implying a potential overlap in function with IL-10, particularly in the context of immune response contraction after Th17-induced inflammation [82]. These findings highlight the multifaceted nature of cytokine interactions and the complex immunoregulatory networks that maintain immune homeostasis [86]. Building on this, our team verified the expression of IL-24 in Th17 cells that were induced in vitro, as well as in uveitogenic Th17 cells derived from EAU mice [5]. We have delineated a regulatory loop wherein IL-17, produced by Th17 cells, retroactively modulates the Th17 cell response. This is achieved through the induction of the inhibitory cytokine IL-24 via the nuclear factor kappa B (NF-κB) signaling pathway [5]. This production of IL-24, stimulated by IL-17, is hypothesized to create a negative feedback mechanism that constrains the secretion of Th17-related cytokines, including IL-17 itself, IL-17F, and GM-CSF, by the upregulation of inhibitory molecules, suppressor of cytokine signalling (SOCS) proteins [5, 42]. Notably, a lack of IL-24 has been associated with an aggravated progression of both EAU and EAE, characterized by an intensified Th17 response [5]. Furthermore, the administration of IL-24, whether systemically or via intravitreal injection, has been shown to mitigate retina inflammation in EAU mice [5, 42]. Additionally, our observations revealed that intravitreal administration of IL-24 in EAU mice led to a reduction in the expression of pro-inflammatory molecules within the retina. This suppressive effect of IL-24 has been replicated in the human ARPE-19 cell line, indicating a broader immunomodulatory role [42]. These findings suggest that IL-24 produced by Th17 cells serves a dual function: it establishes a negative feedback loop that limits the activity of the Th17 cells themselves and also acts on retinal support cells, such as those in the RPE, to alleviate ocular inflammation [5, 42]. Interestingly, IL-24 has also been found to intrinsically suppress Th17 cells by inducing them to produce IL-10. This capability of IL-24 to stimulate IL-10 production operates independently of its interaction with the cell surface receptor on Th17 cells. Instead, IL-24 is translocated to the inner mitochondrial membrane, where it facilitates the preferential accumulation of STAT3 within the mitochondrial compartment rather than the nucleus, thereby modulating the expression of IL-10 [74]. This internal pathway, through which IL-24 induces IL-10 production in Th17 cells, has been demonstrated to play a role in resolving inflammation in the central nervous system (CNS) in cases of EAE. The implications of this mechanism for EAU and retina inflammation, however, will necessitate further research to fully understand.

TGF-β is a critical anti-inflammatory cytokine due to its capacity to suppress various types of immune cells. Additionally, it plays an important role in peripheral tolerance by being essential for the induction of regulatory T (Treg) cells. A recent study demonstrated that Th17 cells express TGF-β1 in an autocrine manner, and TGF-β1-deficient Th17 cells are more pathogenic, exhibiting higher IFN-γ production [87]. This increased pathogenicity has been shown to induce more severe outcomes in adoptive transfer models of EAE and colitis [87]. Further study will be required to investigate the role of Th17 cell-derived TGF-β in the development of uveitis.

**Fig. 2:**
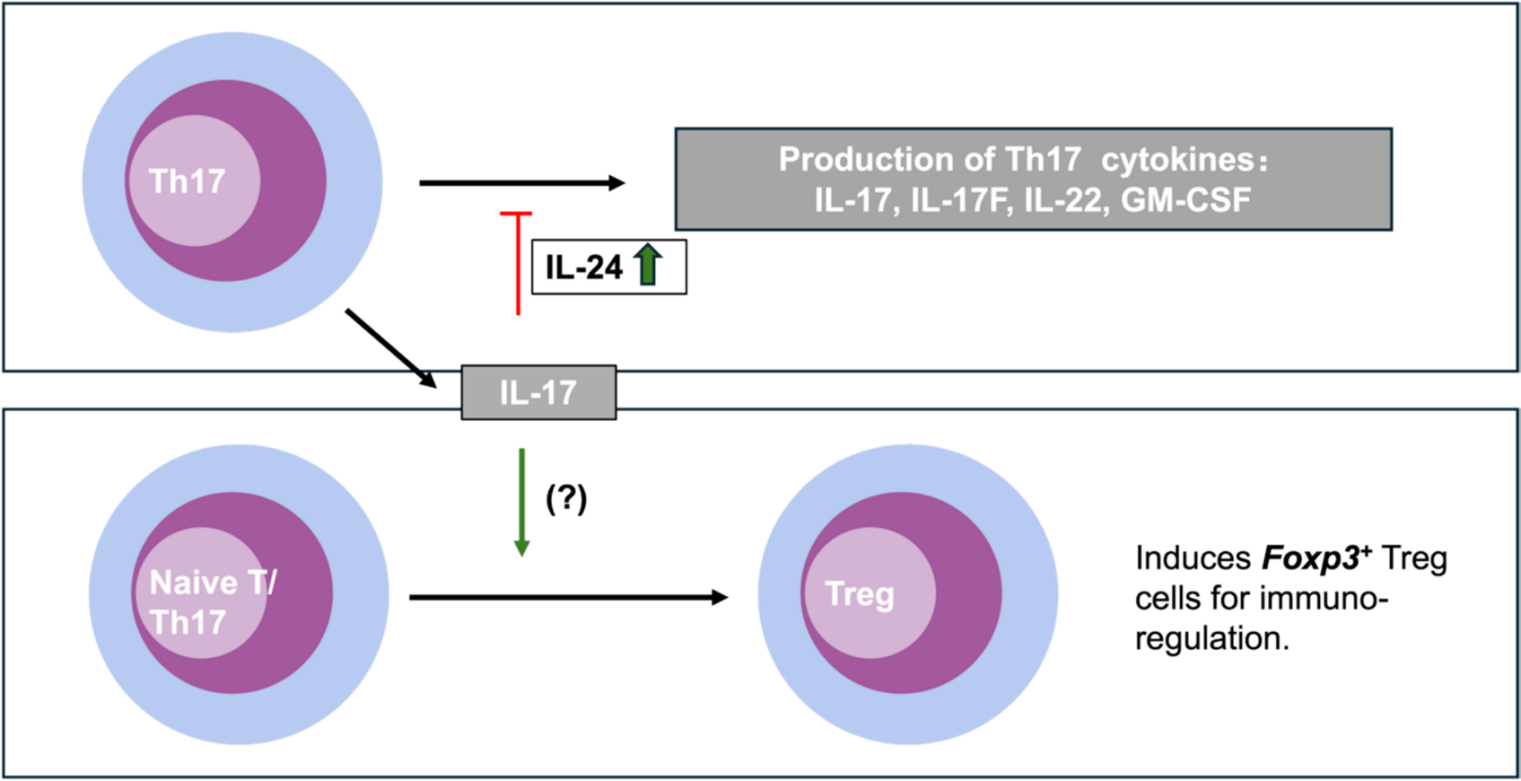
Regulatory roles of IL-17 in immune modulation. IL-17 plays a complex role in immune regulation. Firstly, IL-17 can provide feedback to Th17 cells, triggering the production of IL-24, which then dampens the expression of pro-inflammatory Th17 cytokines. Secondly, IL-17 is involved in promoting the development of Foxp3^+^ Treg cells, although the precise mechanisms underlying this process have yet to be fully elucidated

IL-17 has also been implicated in immune-suppressive functions. Alike IFN-γ, which is well-known for its pro-inflammatory capabilities, its role in immunoregulation has also been established in a number of studies, especially in the context of Th17-mediated retinal inflammation [41, 88, 89]. Similarly, IL-17 was observed to curb the activity of pathogenic Th1 cells, leading to a decrease in intestinal inflammation [78]. It was later found that IL-17 deficiency in Th17 cells results in increased expression of IL-17F, IL-22, and GM-CSF [5, 79, 80], suggesting that IL-17 can act to suppress the production of pro-inflammatory cytokines within Th17 cells. Our subsequent research confirmed that Th17 cells expressing IL-17 can trigger a negative feedback loop that promotes the production of the inhibitory cytokine IL-24 through the activation of NF_K_B signaling pathway, which is crucial for controlling uveitis [5]. In another study on EAU, systemic administration of IL-17 was found to ameliorate disease progression by facilitating the induction of Foxp3^+^ Treg cells, revealing an unexpected tolerogenic aspect of IL-17 in the promotion of Treg cell development [16]. Collectively, these findings suggest that IL-17 cannot only inhibit the pro-inflammatory actions of Th17 cells but also enhance immunoregulation through the promotion of Treg cells and may contribute to the resolution and/or remission of uveitis (Fig. 2).

### Th17 Response in Other Retinal Inflammatory/Degenerative Diseases

Age-related macular degeneration (AMD) is a progressive, degenerative condition affecting the macula, the part of the neuroretina essential for sharp central vision. The precise cause of AMD remains elusive, but chronic inflammation and immune system dysregulation are believed to play key roles in its development [90]. Studies have indicated that PBMCs from AMD patients exhibit an increased presence of IL-17 and IFN-γ producing CD4^+^ T cells compared to those from healthy individuals [91]. These Th17 and Th1 cells have been shown to promote the differentiation of macrophages into a pro-inflammatory M1 phenotype, which is linked to the pathogenesis of AMD [91]. Another potential mechanism implicating IL-17/Th17 in the pathogenesis of AMD is their capacity to promote angiogenesis [92]. The specific mechanisms by which IL-17/Th17 dynamics are altered in AMD patients are not yet fully understood. However, it has been demonstrated that anaphylatoxins C3a and C5a, which are cleavage products of the complement component C5, can modulate the production of IL-1β and IL-6. These cytokines are instrumental in the differentiation of Th17 cells and the subsequent production of IL-17 [92, 93]. Nevertheless, the precise relationship between the Th17 response and AMD warrants further investigation.

Glaucoma encompasses a group of optic neuropathies distinguished by the progressive degeneration of the optic nerve, often in association with elevated intraocular pressure. Traditionally viewed as a disease affecting the optic nerve, recent research suggests that inflammation within the neuroretina also plays a vital role in the development of glaucoma. Inflammatory responses in the neuroretina can precipitate neurodegeneration, leading to damage of the retinal ganglion cells and consequent visual impairment. While direct evidence linking IL-17/Th17 to glaucoma in patients is lacking, Ren et. al. have shown that Th17 cells in patients with glaucoma express higher levels of IL-21, enhancing their ability to stimulate immunoglobulin G (IgG) production compared to Th17 cells from healthy controls [94]. This augmented capacity could be significant in the pathogenesis of glaucoma, considering that the presence of autoantibodies, which can lead to retinal ganglion cell death and optic nerve damage, is a characteristic feature of the disease. Utilizing animal models of glaucoma, Chen et al. demonstrated that disruption of the blood-retina barrier by elevated intraocular pressure permits commensal microflora-primed CD4^+^ T cells to migrate to and accumulate in the retina. This infiltration contributes to the loss of retinal ganglion cells and axonal damage during the prolonged phase of the disease [95]. While this study establishes the involvement of CD4^+^ T cells in glaucoma progression, it also highlights the need for further research to elucidate the specific role of the Th17 response in this context, given that Th17 cells, which are also primed by commensal microflora, have been previously demonstrated to be essential in the development of the spontaneous R161H model of EAU [34].

## Conclusion

In conclusion, the involvement of Th17 cells in uveitis represents a critical focal point for current research efforts. Th17 cells have been identified as key players in the inflammatory cascade associated with uveitis, driving the disease’s pathogenesis by releasing pro-inflammatory cytokines such as IL-17, IL-17F, and GM-CSF. However, insights from EAU studies have unveiled a more nuanced role for Th17 cells. These cells not only initiate and sustain inflammation through their cytokine production but may also facilitate the resolution of inflammation by generating cytokines like IL-10, IL-24, and TGF-β [5, 9, 74, 82, 83]. Interestingly, IL-17 has been implicated in the induction of Foxp3^+^ Treg cells within the EAU model [16], suggesting a complex interplay where IL-17 and Th17 response can contribute to both the onset and the mitigation of ocular inflammation, which may explain the inconsistent results of IL-17 neutralizing agents in treating uveitis [10].

This dualistic nature of IL-17 and Th17 cell responses in ocular autoimmunity is particularly significant, as it calls for a carefully balanced approach in the development of therapeutic strategies that target these cells. The exact functions of IL-10, IL-24, and TGF–β in relation to Th17 cells in EAU are ripe for further investigation, holding the promise of uncovering new therapeutic targets that could finely tune these cytokines to effectively manage uveitis.

## Data Availability

No datasets were generated or analysed during the current study.
